# Re: “Three Calcium Hydroxylapatite‐Based Dermal Fillers Marketed in Mexico: Comparison of Particle Size and Shape Using Electron Microscopy” by Sanchez Rico and Andrade Canto, Journal of Cosmetic Dermatology 2025; 24(3): e70100

**DOI:** 10.1111/jocd.70758

**Published:** 2026-03-07

**Authors:** Sharon Furman‐Assaf, Svetlana Korman

**Affiliations:** ^1^ Medical Writer and Consultant Tel Aviv Israel; ^2^ Dr. Korman Laboratories Ltd. Kiryat Bialik Israel

Dear Editor,

We read with interest the recent article by Sanchez Rico and Andrade Canto (2025), titled “Three Calcium Hydroxylapatite‐Based Dermal Fillers Marketed in Mexico: Comparison of Particle Size and Shape Using Electron Microscopy” [1]. While the study presents valuable observations using scanning electron microscope (SEM) analysis, we respectfully wish to raise several scientific and methodological concerns regarding the interpretation and accuracy of some claims.

First and foremost, from a methodological perspective, the conclusions drawn from scanning SEM imaging of a single sample from a single syringe per product are difficult to generalize. Variability in production lots, storage conditions, and handling could influence particle characteristics. Without replication or batch testing, the findings are best interpreted as preliminary. While this was mentioned as a limitation of the study, we believe that stronger emphasis should have been placed on this constraint, particularly given the definitive tone used elsewhere in the manuscript when drawing comparisons between products. Furthermore, as the authors did not isolate the calcium hydroxyapatite (CaHA) particles from the gel matrix, residues of carboxymethyl cellulose and buffer salts could compromise the appearance of the particles on SEM images.

CaHA microspheres are fabricated from submicron particles consolidated into 25–45 μm spheres and sintered into dense ceramics. During sintering, the primary particles coalesce into grains a few microns in size, creating a dense ceramic body. Hence, the material is expected to have a slightly rough surface. The SEM image of Sample 1 particles (HArmonyCA) indicates they may be coated with hyaluronic acid, as the authors themselves suggest in the discussion. This coating differentiates Sample 1 from Samples 2 (Radiesse) and 3 (Hydroxyfill), making direct comparisons of surface smoothness inappropriate. There is no evidence whether this coating is beneficial for the clinical performance of the material. Moreover, the coating will be resorbed early, and the underlying surface of the bare ceramics is expected to be rough. Indeed, on the SEM images, some uncoated particles in Sample 1 appear rougher than those in Samples 2 and 3.

Furthermore, the SEM images provided are too low in resolution to distinguish whether the observed roughness arises from coarse ceramic grains or remnants of the gel matrix. While SEM is useful for morphological assessment, drawing conclusions about clinical superiority from particle shape, such as smoothness or roundness, lacks in vivo validation. Without supporting clinical or histopathological data, such claims remain speculative.

The authors assert in the discussion that “The fragmentation observed in Hydroxyfill, creating rough surfaces that interact with immune cells, raises concerns about heightened inflammatory responses”, without providing the evidence for such a claim. The authors cite Laeschke's review [2] to support their claim that the surface structure of microparticles significantly affects biocompatibility: smooth surfaces tend to promote better tissue integration and milder inflammation, while rough or irregular surfaces can trigger stronger and more unpredictable immune responses. However, Laeschke [2] does not provide a citation to support this claim in the text or in the reference list in relation to CaHA fillers. Their reference list includes the study by Ozgentaş et al. [3], who histologically examined the fate of subcutaneous implantation into rats of the collagen implants Zyderm II and Zyplast, as well as Vicryl, and chromic gut after one year, and showed no difference in long‐term persistence between the two collagens while both Vicryl and chromic gut implants caused an intense inflammatory response at 1 month. Another cited reference, Taylor and Gibbons [4], compared the soft tissue response to textured versus smooth polytetrafluoroethylene (PTFE) implants in rats. Surface texture was precisely created using ion beam etching, producing conical projections on the PTFE without altering its chemical properties. They showed that surface texture, rather than chemistry, is a key determinant of the soft tissue response to PTFE implants, with textured surfaces promoting more favorable biological interactions and reducing fibrous encapsulation. An additional study cited by the same review [5], examined the effect of the size, shape, and surface area of polymethylmethacrylate (PMMA) particles on the type and intensity of the inflammatory response. They showed that while larger spherical particles caused a greater inflammatory response overall, small particles induced greater tumor necrosis factor levels, suggesting different cellular mechanisms for cytokine release. Therefore, Sanchez Rico et al.'s claim [1], which is based on Laeschke's review [2] does not directly support the assertion that smoother CaHA microparticle surfaces inherently lead to better biocompatibility in dermal fillers; rather, the cited studies focus on other implant materials and demonstrate that controlled surface texturing can, in some contexts, enhance tissue integration and modulate inflammation, contradicting the generalized assumption that smoother is always better.

Moreover, a more recent histological study conducted 2 months after the injection of Radiesse into the deep dermis of 5 women scheduled for redundant abdominal skin removal, showed no immune activation [6]. Karatas et al. [7] found good tissue integration and limited inflammation with CaHA microspheres ranging in size from 20 to 60 μm (80% were 30–50 μm) dispersed in a carrier phase composed of carboxymethyl cellulose, glycerol, and water for injection. The CaHA particle surfaces had pores and cavities between 2 and 5 μm, implying morphology alone is not predictive of response.

The authors also claim that the effect of hyaluronic acid products lasts only for 4 to 6 months, while many studies have shown that the effect of crosslinked hyaluronic acid products is up to 18 months, depending on the crosslinking method and product [8, 9, 10, 11, 12, 13, 14, 15].

Finally, we have noted that the authors have declared no conflicts of interest. However, according to Dr. Gilberto A. Sánchez Rico's website, he is a speaker for Allergan, Merz, and Galderma (https://onelineclinic.com/en/inicio‐english/).

In conclusion, while the study by Sanchez Rico and Andrade Canto [1] adds to the growing literature on filler morphology, several claims require clarification to avoid misrepresentation. We encourage the journal to consider a correction or author response to address these issues and support scientific integrity in the field of cosmetic dermatology.

## Author Contributions

S.F.‐A. wrote the original draft and revised the letter. S.K. critically read and revised the letter. Both authors approved the final version.

## Funding

The authors have nothing to report.

## Ethics Statement

The authors have nothing to report.

## Consent

The authors have nothing to report.

## Conflicts of Interest

Sharon Furman‐Assaf received funding from Dr. Korman Laboratories. Svetlana Korman is Dr. Korman Laboratories' medical director.

## Linked Articles

This article is linked to Sanchez Rico and Canto paper. To view this article, visit https://doi.org/10.1111/jocd.70100.

## Data Availability

Data sharing not applicable to this article as no datasets were generated or analyzed during the current study.
